# “Knock Knock”: a qualitative study exploring the experience of household contacts on home visits and their attitude towards people living with TB in South Africa

**DOI:** 10.1186/s12889-020-09150-1

**Published:** 2020-07-02

**Authors:** Farzana Sathar, Kavindhran Velen, Meaghan Peterson, Salome Charalambous, Candice M. Chetty-Makkan

**Affiliations:** 1 The Aurum Institute, 29 Queens Road, Parktown, Johannesburg, Gauteng 2193 South Africa; 2 Emory University Rollins School of Public Health, Atlanta, GA USA; 3 School of Public Health, University of Witwatersrand, Johannesburg, Gauteng South Africa

**Keywords:** Qualitative, Tuberculosis, Household contact tracing, Active case-finding

## Abstract

**Background:**

Household contract tracing (HHCT) is an important strategy for active tuberculosis case finding and offers an opportunity for testing of other diseases such as HIV. However, there is limited data on the patient-centered approach to HHCT. Our study aimed to describe experiences and preferences of household contacts (HHCs) for HHCT.

**Methods:**

We conducted a qualitative study in Rustenburg, South Africa from September 2013 to March 2015. Twenty-four HHCs (≥18 years) had audio-recorded in-depth interviews. We used an inductive thematic analysis approach to develop themes. We made an a priori assumption that we would reach saturation with at least 20 interviews.

**Results:**

There were 16 (66.7%) females (median age = 36 years) and eight (33.3%) males (median age = 34 years). Two themes developed: (i) Positive attitude of HHCs towards TB services provided at home and (ii) HHCs relationship to and acceptance of people living with TB (PLTB). The first main theme emphasized that HHCs appreciated the home visits. Participants preferred home visits because they had negative experiences at the clinic such as delayed waiting times and long queues. HHCs supported the screening of children for TB at home. Participants suggested that the research staff could expand their services by screening for diabetes and hypertension alongside TB screening. In the second main theme, there was a sense of responsibility from the HHCs towards accepting the diagnosis of PLTB and caring for them. A sub-theme that emerged was that as their knowledge on TB disease improved, they accepted the TB status of the PLTB empowering them to take care of the PLTB.

**Conclusions:**

HHCs are supportive of HHCT and felt empowered by receiving TB education that ultimately allowed them to better understand and care for PLTB. HHCs were supportive of screening children for TB at home. Future HHCT activities should consider raising community awareness on the benefits of TB contact tracing at households.

## Background

In 2018, the Global TB report stated that South Africa had a total tuberculosis (TB) incidence rate of 520 per 100,000 and a HIV-positive TB incidence rate of 306 per 100,000 [1]. Despite a gradual decline in TB incidence over recent years, focused efforts are required to close gaps along the cascade of TB care to reach the End TB targets. In response, the World Health Organization (WHO) launched a joint initiative with The Stop TB Partnership and The Global Fund to Fight AIDS, Tuberculosis and Malaria titled “FIND. TREAT. ALL.” [2] which encouraged innovation and targeted approaches for finding and treating TB patients to identify the 40 million people required to reach the 2018–2022 targets [1].

One key strategy, active case finding, entails a systematic approach to identifying individuals with TB, typically targeted towards high-risk groups such as household contacts (HHCs) of people living with TB (PLTB) [3]. Household contact tracing (HHCT), a form of active case finding, involves healthcare workers visiting the homes of PLTB to evaluate their HHCs for TB [2]. HHCT overcomes barriers such as the need for patients to present at healthcare facilities for TB screening [4], but also enables early identification of undiagnosed TB, ultimately reducing transmission, morbidity and mortality [5–8]. Studies in South Africa have demonstrated success in detecting high proportions of undiagnosed TB and HIV among HHCs, such as the “Ribolola” Programme in the North West Province of South Africa [9, 10].

The additional value of HHCT is the ability to evaluate children for TB or refer them to the health facility for investigation; studies in Africa and Asia have demonstrated success in doing this [11–17]. Studies in Swaziland and Uganda found that prioritizing children in HHCT is a major contribution to case finding [11, 13]. In The Gambia, among 4000 child contacts (< 15 years) screened at home, 1.6% had TB, of which 40% were asymptomatic [14]. This highlighted the importance of screening all child contacts in the household regardless of symptom manifestation.

Although there is strong evidence for HHCT to close gaps in TB case detection, the strategy relies entirely on the assumption that HHCs will allow healthcare workers into their homes to perform TB screening evaluation. A study in Vietnam examining theoretical acceptability among HHCs showed high approval rates up to 88% [18].

While initial data suggested HHCT is feasible in the context of South Africa [9], we know little about the experiences and preferences of HHCs that may influence the optimization of this intervention. In addition, there is limited knowledge on the relationship dynamics of HHCs and PLTB that may influence the acceptability of home visits for TB screening. Thus, if we are to realize the End TB targets, pursuing a patient-centered approach will require us to understand perceptions and preferences to the delivery of key interventions such HHCT.

## Methods

This was an exploratory qualitative study conducted among HHCs from September 2013 to March 2015. HHCs were enrolled from a larger parent study conducted in Rustenburg, Bojanala district, South Africa. The aim of the parent study was to compare the yield of TB among HHCs of Rifampicin-resistant (RR-TB) and drug-sensitive tuberculosis (DS-TB) patients. In 2016, the Bojanala district had a population of 1,657,148 people [19] where majority of the population (64.8%) was between 15 and 64 years old and 30.3% under 15 years old. Among these, 32.5% completed secondary school and 6.5% had a tertiary education. The mean household size was 2.7 per dwelling, with around 30% in informal dwellings. Health care is available with 115 primary healthcare clinics in Bojanala and 21 of these in the Rustenburg sub-district (population of 549,575) providing TB screening and testing services [20]. In 2015, the Bojanala district had a TB incidence of 419 per 100,000 [21].

The parent study purposively sampled PLTB (DS- or RR-TB patients) identified through routine testing conducted by the National Health Laboratory Service (NHLS); these patients were subsequently contacted by study staff and invited to participate in the study. Consenting PLTB had their households visited at a pre-arranged time and their HHCs evaluated for TB. HHCs ≥5 years old were evaluated for TB using symptom screening (cough > 2 weeks, night sweats, fever, unintended weight loss), while those < 5 years were referred to the clinic for evaluation.

Data collection for the qualitative component took place a month after the initial household visit was completed, as per requirements of the parent study. The aim of our qualitative study was to gain an understanding of HHCs preferences and perceptions towards HHCT. We selected HHCs to participate using convenience sampling from a sample frame of participants enrolled into the parent study. We telephonically contacted the sample of HHCs (≥18 years old) and invited them to be interviewed. We interviewed 24 HHCs at their homes using a structured in-depth interview guide that we developed specifically for the purposes of this study (Appendix 1). Although a structured interview guide was used, probing on the questions allowed new data to develop from the participant responses. The following topics were explored using the guide; household contact knowledge of TB, preference for clinic visits versus home visits, views around HIV testing and other services.

Male and female research staff with a background in social science and prior qualitative experience conducted the participant interviews. Research staff received qualitative training prior to conducting participant interviews to maximize and standardize discussions with patients. HHCs provided informed consent prior to the interview, permission to audio-record and use the information collected as direct quotes in the publication. Each participant had one in-depth interview and there were no repeat interviews. Only one study staff member was present during the interview. The length of the interviews ranged between 15 and 45 min and all sessions were audio-recorded. The languages used during the interviews were Setswana and Sotho; we transcribed the interviews verbatim and then academically qualified research staff with at least an Honour’s level academic qualification translated it into English. We used the COREQ (COnsolidated criteria for REporting Qualitative research) checklist for writing this manuscript (Supplement 1).

### Data analysis

We coded English transcripts only. The data coders did a quality check on the transcripts to ensure the removal of all personal identifiers and that there was consistency between audio-recordings and transcripts. Two data coders manually coded the data (FS and MP). Both data coders have Master’s level qualifications. The senior author is an experienced qualitative researcher with a doctoral degree and supervised the data analysis (CM). The data coders manually coded a sample of the transcripts and the senior author (CM) qualitatively and quantitatively checked inter-rater reliability between the coders. Independent fine coding of the transcripts only took place after reliability of coding was above 90% consistent between the data coders. We used inductive thematic analysis to develop themes. Prior to the study, we assumed that the planned number of interviews would help us reach saturation. We also included both genders to account for variation in responses. An excel spreadsheet was used to monitor the diary of steps that were used during the manual coding process. We attach all de-identified transcripts as supplementary information (Supplement 2).

## Results

We telephonically contacted individuals from 15 households to participate in the in-depth interviews. Among these, individuals from 11 (73.3%) households agreed to participate and 4 (26.7%) households did not want to participate in the interviews. There was a mean of 2 contacts per household, similar to the mean household size of the area population. We enrolled 24 HHCs for qualitative work. There were 16 (66.7%) females: median age in males 34 years, and females 36 years. There were 4 (16.7%) self-reported HIV positive HHCs. No other demographic information was collected. We derived two major themes from the analysis; (i) Positive attitude of HHCs towards TB services provided at home and (ii) HHCs relationship to and acceptance of PLTB.

### Main theme 1: positive attitude of household contacts towards TB services provided at home

All HHCs appreciated the home visits and did not have a problem with research staff visiting them. Participants were not concerned about others in the community being aware of the home visits through use of a branded vehicle by the research team. Participants preferred home visits because they had negative experiences at the clinic such as delayed waiting times and long queues. TB screening services offered at home was also beneficial to children as they were less likely to go to the clinic. The quotations from three participants below describes their satisfaction with the home visit, especially related to screening their children for TB.

*“I really liked the job they did for us; they made things easier for us because had they not come here I wouldn’t have {had} my children tested for TB, I would still be sitting here at home. I appreciate that they came here to test the children because I was concerned about their wellbeing. I am really happy about that because even my mother was tested and she hardly goes to the clinic to get tested. I am really happy for what they did for us … I was happy that they came to test the children for TB.” (ID 20)*

*“We just wanted those people to teach us new things, we did not have a problem at all.” (ID 21)*

*“All in all, it was good … An effort was made to get closer to us so that we can get tested; I think it was something good.” (ID 22)*

Participants did not feel inconvenienced by the home visits and said that the research staff could visit them any time.

*“No I’ve no problem, you can come day, night, at anytime.” (ID 1)*

*“Any day of the week because I am unemployed. Any time is okay with me.” (ID 17)*

However, participants suggested that the research staff could expand their services by screening for other illnesses such as diabetes and high blood pressure. They also felt that the broader community should be reached as they could benefit from TB screening at their homes, since it is a helpful service.

*“It is very important. You should encourage people to test, if a person is not feeling well they should test for TB, diabetes, high {}{blood} and all other diseases.” (ID 24)*

*“I don’t know, if you were to make a notice or maybe something like, I don’t know, have you ever seen those Coca Cola people on a lorry speaking on speakers?” (ID 4)*

### Main theme 2: household contacts relationship to and acceptance of PLTB

There was a sense of responsibility from the HHCs towards accepting the diagnosis of their PLTB. However, it seemed that this feeling of taking care of their family member who was diagnosed with TB, was more out of a sense of obligation. Quotations from two participants describe their feelings towards their family member:

*“So I’ve got a son with a TB … For now he’s okay and let me say that I’m dealing with that because I {} {have} no choice.” (ID 1)*

*“But the problem will be that I have a partner and I have asked him to accommodate the patient and there is no way I can abandon the sick patient.” (ID 3)*

### Subtheme: knowledge gained from the home visits that influenced acceptance of PLTB

Although most HHCs described accurate knowledge of TB symptoms because of their experiences living with PLTB, they were still uncertain of how to take care of them. Participants felt that the information on TB disease and treatment provided during the home visits helped them to accept the TB status of the PLTB. Quotations from four participants below describe how the home visits empowered them to take care of the PLTB:

*“The way you talk to me{} about that encourage {s} me … I like {it} because you give me hope to … to … to carry on … Because that’s the big help for me, for now I’ve got that courage because since the people come there I’ve got that{} courage because I know I’m not alone this time. There are people who{}{are} going to help me … You see that the things that make {} {you} say ‘okay I feel very happy because I got that courage now’, you are there, coming there to help me. I got that courage, okay there is somebody who {is} thinking about me, thinking about my situation.” (ID 1)*

*“Actually I thought that he was dying because he lost weight, too much weight yah so it {} hurt not knowing what is killing him and all that. But when I found out that it has treatment and it has a cure that gave me hope.” (ID 2)*

*“By coming here to talk to us and also they helped that person who had TB to accept his condition and then he helped us to understand where we all stand.” (ID 17)*

*“Okay, they explained to us that we must keep his room clean and give him appropriate food. I had always thought that when a person has TB you cannot share things such as plates with them, but after he was given the treatment we were told that we could share plates with him because they explained to us that TB is a bacterium on air.” (ID 22)*

## Discussion

HHCs had a general positive attitude towards HHCT and home visits were preferred in comparison to attending clinics. HHCs gained valuable knowledge and understanding about TB disease that ultimately lead to their acceptance of a family member effected by TB. Initially HHCs felt obligated to care for their family member who had TB but later felt empowered by the knowledge gained from the home visit. Additionally, HHCs felt that the home visits provided an opportunity for TB screening for their children or referral to the health facility for investigation.

Our study findings are consistent with other qualitative studies in Africa and South-East Asia, which found that community members supported the concept of HHCT [11, 18, 22, 23]. A common reason for appreciation of TB services at home was it removed barriers of access and cost. Community members did not have to travel long distances to access clinic services. Despite the overall positive attitude in these studies [11, 18, 22, 23], there have been examples in South Africa where HHCs were not comfortable with home visits from research staff, specifically concerned with associated stigma and the opinions of their community members [22]. In contrast, our study participants did not express this same concern implying that there was no stigma attached to HHCT.

The willingness of HHCs to want more education on TB in our study is consistent with other studies in South Africa, Cambodia and Columbia, where participants wanted healthcare workers to educate their whole community on TB [22–24]. Such evidence has demonstrated the value of HHCT as a platform for disseminating information on TB. TB information in the form of pamphlets in the local language with infographics is available in theory, however access to the web in this population is limited. By having better knowledge of TB, HHCs were able to cope with caring for their PLTB, despite an initial sense of obligation. They felt hopeful and encouraged by having professionals come to their home to talk to them about TB. HHCs knowledge about TB disease and treatment enabled better acceptance of their PLTB, improving their overall care for them. Therefore, by improving people’s knowledge of TB, we are empowering them to know when to seek healthcare.

Despite the referral of children to the clinic for TB screening during the parent study, HHCs seemed to prefer that TB screening of children take place at the household. However, the HHCs may not be aware of the complexities on screening children for TB at home. Children under five are a high-risk group and even after exclusion of TB disease, Isoniazid preventive therapy (IPT) is provided [25]. Therefore, additional education around screening children for TB will be beneficial.

When considering a patient-centered approach to HHCT, this study showed that HHCs want to be empowered with knowledge on caring for PLTB, TB screening of children and provision of services for other diseases. HHCT may thus provide a platform for a more holistic approach to healthcare beyond TB screening alone. Expanding the services of HHCT may enable case finding to transcend the household and reach other members of the community by creating awareness and encouraging TB screening.

### Strengths

The overall strength of the study is that it provided a HHC perspective that could help refine approaches to HHCT. We included a diverse group of participants based on age and gender that provided a balance of views and perspectives on household provision of TB care. We conducted interviews a month after the household visit to minimize recall bias of their experience.

### Limitations

Staff who conducted the HHCT and the participant interviews were from the same organization. Participants may have felt obligated to respond positively, suggesting a level of social desirability bias. However, we attempted to overcome this limitation by having different research staff conduct the interviews instead of those who conducted the home visits. Although we conducted 24 in-depth interviews, we did not revise probes during data collection. However, during data analysis we found that themes started to recur based on the in-depth interview guide. We did not explore perceptions of parents regarding the referral of children < 5 years old to the clinic for evaluation.

## Conclusions

HHCT is a promising intervention for active case finding, however shifting to a patient-centered approach will elevate its application and effectiveness. By exploring the experience of HHCs, we can improve the delivery of healthcare at the household level. Our study found that there is a willingness for TB screening of children at the household level so HHCT should consider strategies for evaluating children within the household; this may help with reported attrition associated with referrals. It may also be beneficial to expand the scope of HHCT by including an education component to TB screening at home and to explore whether awareness and knowledge on TB disease targeted at HHCs encourages the HHCs to screen for TB.

## Supplementary information

**Additional file 1.**

**Additional file 2.**

## Data Availability

Data and materials are available and requests for these can be sent to the corresponding author.

## Abbreviations

DS-TB: Drug-sensitive tuberculosis
HHCs: Household contacts
HHCT: Household contract tracing
IPT: Isoniazid preventive therapy
NHLS: National Health Laboratory Service
PLTB: People living with TB
RR-TB: Rifampicin-resistant tuberculosis
TB: Tuberculosis
