# Supplementary material for: “Knock Knock”: a qualitative study exploring the experience of household contacts on home visits and their attitude towards people living with TB in South Africa
Source: BMC Public Health. 2020 Jul 2;20:1047. doi: 10.1186/s12889-020-09150-1 (PMC7331256; doi:10.1186/s12889-020-09150-1)
Supplement: Supplementary file 1 — Additional file 1. [file 12889_2020_9150_MOESM1_ESM.pdf]

QUALITATIVE RESEARCH IN-DEPTH INTERVIEW GUIDE

Introduction

- Greetings
  - Introduce yourself to the informant (your name and where you work)
  - Explain to the informant that we are doing a project to see what is the best way to visit homes who have a family member that is found with TB so that they can also be checked for TB and HIV
  - For this project we will be asking them questions about TB and HIV. We want them to be as open an honest when answering and tell us what they like and dislike us to do when we visit their homes especially when someone in their home has TB.
- Confidentiality
  - All information collected during the course of this project will be kept safe and secret.
- Study Identifiers
  - We will not use your name or any other identifying information and everything that you say will only be used for research purposes

Interview Guide

1. How are you doing today?
2. Household Contact Tracing
   a. Perceptions/Preferences
      i. Have your heard about a disease called tuberculosis? What have you heard?
      ii. Do you know how tuberculosis can be spread? If yes, tell me more.
      iii. Can you talk about TB in your house? If yes or no, what are some of the reasons?
      iv. What did you like about us visiting your house for this project?
      v. What did you not like?
      vi. How can we make it better for you or other people in your community when we visit you for this project?
      vii. What do you think your neighbours would say if they saw our vehicle at your house? Does this make you feel good or bad? Would it be better or worse if the vehicle didn't have signs on it?
      viii. Would you like us to talk to you in the project vehicle on in your house; what are your reasons for saying this?
      ix. Did you enjoy our visit to your household; if yes or no, what are some of the reasons for saying this?
      x. Would you have preferred to go visit a clinic of have someone visit you at home; what are some reasons for saying this?
      xi. When is the best time for us to visit you-at your home to provide health services? During the day or in the afternoon after work?
      xii. When health staff visit you at home, are there any other things you would like them to help you with? Blood pressure or diabetes check?
   b. HIV testing

i. Tell me about HIV and how does it affect your community?
ii. How do you feel about us offering you HIV testing in your house? Do you think it is a good idea?
iii. Did you test for HIV recently? How did you decide to test?
iv. How did you feel about the process?
v. What did the result mean to you?
vi. What were the difficulties that you had about testing in your house?
vii. How would you feel if we offered more than one type of HIV test to choose from at your household? (Oral vs. rapid vs. ELISA)
viii. Is there anything you think could be done differently when health workers visit your house?

3. Would you allow health service staff to visit you household again in the future for household contact tracing of TB; why?
4. Thanks for participating and can we contact you again if I have any further questions or need further clarification?

END

TIPS FOR INTERVIEWING

- Do not begin interviewing right away
- Friendly greeting and explanations
- Listen and express interest in what the informant tells you
- More of a friendly conversation
- Not a strict question & answer exchange
- But remain neutral: don't approve or disapprove
- Try to encourage informant to expand on their answers and give as many details as
- possible
- If the informant's has a tendency to abbreviate answers rather use "describe," "tell me about"
- Do not move on to a new topic until you feel you have explored the informant's knowledge on the question at hand
- Let informant's answers determine the direction the interview takes (keeping within topics of interest)
- Use informant's own language to ask new questions; do this as you learn informant's language
- This encourages informants to speak to you in their own language
- Learn how to re-phrase/re-think questions
- Avoid using "why" questions as much as possible as informants will tend to think the answer they have given you isn't the correct one and will proceed to give you socially acceptable answers
